# Ambulance professionals’ adaptations in prehospital services: a critical incident study

**DOI:** 10.1186/s12873-025-01309-6

**Published:** 2025-08-15

**Authors:** Cecilie Erga, Stephen J. M. Sollid, Karina Aase

**Affiliations:** 1 SHARE- Center for Resilience in Healthcare, Department of Quality and Health Technology, Faculty of Health Sciences, University of Stavanger, Kjell Arholms Hus, Kjell Arholms Gate 43, Stavanger, 4021 Norway; 2 Department of Research and Development, Norwegian Air Ambulance Foundation, Oslo, Norway

**Keywords:** Adaptations, Ambulance professionals, Prehospital services, Communication

## Abstract

**Background:**

The working environment for ambulance professionals in prehospital services is complex, dynamic, and associated with a high degree of unpredictability. It is therefore essential that ambulance professionals adapt to provide high-quality and safe care, yet the research literature on how they successfully adapt in their everyday work remains sparse. The aim of this study is to address this knowledge gap by exploring adaptations in the context of prehospital services, through ambulance professionals’ descriptions of successful missions.

**Methods:**

A qualitative descriptive study was conducted using the Critical Incident Technique methodology for data collection and analysis, the latter through the processes of re-storying and cross-incident analysis. Twenty semi-structured individual interviews were conducted between October 2023 and May 2024 with ambulance professionals, including licensed ambulance medical technicians and paramedics with dual licensing or other additional medical licenses across four ambulance stations in Norway with contrasting geographical locations.

**Results:**

A wide range of successful adaptations were described by the ambulance professionals and grouped into seven core themes: (1) Adaptations in mission planning; (2) Practical adaptations; (3) Time-critical adaptations; (4) Personal adaptations; (5) Task-focused adaptations; (6) Adaptations in stakeholder coordination; (7) Adapting to patients and informal caregivers.

**Conclusions:**

This study provides insight and new knowledge about successful adaptations in prehospital services and illuminates the variety of adaptations ambulance professionals make in different contexts. Trust is an underlying feature for successful adaptations, while communication is the overall predominant feature, especially vital in stakeholder coordination and decision-making processes impacting team efforts and mission efficiency. Further research should provide insight into cross-occupational and cross-stakeholder collaborative processes.

**Supplementary Information:**

The online version contains supplementary material available at 10.1186/s12873-025-01309-6.

## Background

Ambulance professionals (APs) in prehospital services care for patients outside the hospital, where the environment is complex, dynamic, and highly unpredictable. Their everyday work involves events and conditions that cannot be fully foreseen or planned for, requiring APs to make decisions that support patient safety [1–8]. APs typically work in small teams consisting of two or three APs who regularly collaborate with other AP teams and healthcare professionals [9]. As well as collective efforts, individual initiatives are important for operational continuity and safe patient care [10].

In this study, we explore adaptations made by APs working in ambulance cars, as they often encounter conflicting goals, and the core operational challenges and reconciliations of these goals involve issues of choice, decision-making, and adaptation [11]. The term ‘adaptation’ refers to a specific mechanism or action in response to a particular challenge or change [12], involving explicit or implicit trade-offs, where decisions are made consciously or unconsciously. Decision-making processes involve clinical reasoning in which information is collected, evaluated, and used, to make assessments and decisions about the course of the patients’ medical care. These processes are not linear, and also comprise additional assessments and considerations regarding the patient’s individual needs, such as their perceived, physical or existential discomfort [13, 14]. Strategies for adaptations that support decision-making processes are largely built on the job, based on previous knowledge and experiences [11]. Different contexts and patients require different actions, and it is essential that APs adapt accordingly to provide high-quality and safe care, which is the case in most prehospital missions [15]. Within the prehospital context, current research investigates clinical reasoning and decision-making [13, 16–18], but research on adaptations in the prehospital service is lacking [5, 19]. Current research on adaptations is mainly conducted in hospital contexts [20–22], at organizational or system levels [12] or focus on specific diagnoses, rather than adaptations in work or mission processes [23–27]. A more comprehensive lens is therefore needed for untangling adaptations carried out by APs in prehospital services [7]. The aim of this study is to address this knowledge gap by exploring adaptations in the context of prehospital services, through ambulance professionals’ descriptions of successful missions. To achieve this, we have worked according to the following research question: How are successful adaptations during ambulance missions described by APs? This was explored by asking APs to describe ambulance missions they experienced as successful, where adaptations were uncovered through data analysis.

## Methods

### Design

This qualitative study utilized the Critical Incident Technique (CIT) [28] as both interview and data analysis method, guided by Watkins et al. [29]. As described by Flanagan, CIT is *“a flexible set of procedures for gathering certain important facts concerning behavior in defined situations”* [28, p.9] which are particularly suitable for studying human behavior and intent for action, with findings derived from participants lived experiences [30], such as APs’ experiences of how they conduct adaptations during ambulance missions. In CIT, a ‘critical incident’ does not mean that the experiences or events must be critical, or in the case of this study, must involve critically injured or ill patients, or critical incidents in terms of adverse events for patients or APs. Rather, a ‘critical incident’ is an episode, event or situation that subsequently triggers an action which has a significant impact on the final result (i.e., successful adaptation) [30]. We have applied Flanagan’s original term ‘critical incident’ [28], as recommended by Viergever [31]. CIT involves a five-step process of (1) establishing the aim of the activity being studied, (2) make plans and specifications, (3) data collection, (4) data analysis, and (5) data interpretation and reporting results [28, 29, 31, 32]. The steps are described in more detail in Schluter, Seaton, and Chaboyer [33]. This study followed the COREQ 32-item checklist for reporting qualitative research [34].

### Setting

The study was conducted in the prehospital services of two Norwegian hospital trusts, across four ambulance stations with contrasting geographical representations. Two stations were in urban areas, with approximately 15,000 and 3,000 yearly missions, respectively, and two in remote areas, each with approximately 3,000 yearly missions. Norway is a long-stretched country with challenging geography and climate. The population of around 5.6 million is mainly centered around the larger cities, but approximately 17% live in decentralized parts of the country [35]. Combined with the right to equitable healthcare, this presents challenges to the prehospital services, and influences response time, on-scene time and transport durations [36]. The Norwegian emergency medical services system has been extensively described in a recent study [37]. In Norway, APs generally have two levels of education. The majority are trained at a basic level as ambulance service technicians (ASTs), with two years of vocational school and a two-year apprenticeship before obtaining the AST qualification. Since 2014, a three-year bachelor’s degree in paramedicine has also been available. In addition, some prehospital services offer internal education as an add-on to the basic AST training. Until recently, a 60 ECTS paramedic training program was available for ASTs, but this has now been replaced by the Bachelor of Science in Paramedicine. A limited number of ASTs also hold a bachelor’s degree in nursing, a combination regarded as equivalent to a bachelor’s degree in paramedicine [38].

### Recruitment

Participants were invited to participate via their station leaders, who presented the study and the opportunity to participate approximately two months before data collection. Two weeks before the interviews, the leaders provided potential participants with a project information sheet detailing the study’s purpose, what participation would involve, the first author’s contact information, and that they had the right to withdraw from the study at any point without negative consequences. The leaders subsequently recruited and coordinated a total of twenty individual interviews.

### Sample

Participants were purposively selected according to availability and the inclusion criteria of having a minimum of three years of working experience in prehospital services. The experience criterion was set at three years to ensure that participants had adequate occupational insight to provide rich mission descriptions. Participants consisted of thirteen men and seven women between the ages of 27 and 58 years, with an average of 15 years (range 5 to 20 years) of experience in prehospital services. The participants were either licensed ASTs or paramedics with dual licensing or other additional medical licensing (e.g., nursing).

### Data collection

The first author (CE) conducted twenty face-to-face semi-structured individual interviews in private rooms at the ambulance stations where the participants worked. An interview guide (Additional file 1. Interview guide) was developed and tested with two experienced professionals currently involved in the education of APs. No relationships between the author and participants were established prior to the study commencement. The interviews were either conducted before the APs started their shifts or during their duty time that day, and some participants came in during their free time to do the interview. Either way, participants received their normal hourly wages for taking part in the study. In cases where interviews took place before or during their shifts, the station leader had coordinated the time for the interview so that the station was adequately staffed during the interviews by overlapping shifts. The APs were therefore not on call during the interviews, and the ambulance stations remained adequately staffed. There were no dropouts from the study.

Each interview began with a presentation of the interviewer (first author), including work and research experience, and reasons for conducting the study. It was emphasized that the first author had no experience with prehospital services. Thereafter, the study’s aim was explained, followed by participants being asked to share, in detail, a relatively recent self-selected work mission they had experienced as particularly successful, emphasizing that it was the participants’ experiences and descriptions of the mission that were of interest, not the outcome of the mission itself in terms of patient outcomes or response times. The participants had been informed about this request via their leaders before the interviews to allow time to think of a potential mission in advance. Furthermore, it was specified that the mission’s criticality was unimportant, but that it should involve some level of patient treatment, not merely patient transport. This was specified according to the focus of exploration, i.e., successful adaptations during ambulance missions. Participants were encouraged to choose a relatively recent mission to better be able to accurately provide a detailed account of its content. A few chose to share missions from further back, which they justified by explaining that the missions had a strong impact on them. Since CIT interviews are based on participants’ self-selected events (i.e., missions), this was not “corrected”. If necessary, the interviewer asked follow-up questions for clarification or elaboration during the interviews. Toward the end of each interview, the interviewer summarized the participants’ narratives, emphasizing a mutual understanding of the substance and essential elements important to the participants’ experience in relation to the research question. The interviews took place between October 2023 and May 2024 and lasted approximately sixty minutes. The interviews were audio recorded and transcribed using Nettskjema, a digital solution developed and hosted by the University of Oslo [39], and subsequently re-transcribed verbatim and de-identified (CE).

### Data analysis

Qualitative CIT data analysis comprises two distinct processes: re-storying and cross-incident analysis [29]. In the process of re-storying, the participant’s story illustrates what is learned about the phenomena of interest, i.e., successful adaptations, and what it looks like in an organization, i.e., prehospital services. A narrative inquiry-based approach was used, where all elements of the participants storylines were rearranged and formed into a chronological narrative. Other than anonymization, none of the words or phrases used by the participants were changed or excluded. Through several readthroughs, a headline for each narrative was formed, capturing the essence of the narratives (e.g., *“Our relationships*,* skills*,* and knowledge of the procedures contributed to good solutions”*), and critical incidents were identified (e.g., *“While waiting for the other ambulance*,* we prepared everything that could be prepared”*). Critical incidents are elements of the narrative that undoubtedly made a significant contribution to the aim of the study [28], i.e., successful adaptations during ambulance missions. The data material consisted of 450 critical incidents in total, which exceeds the lower limit of incidents traditionally considered satisfactory in a CIT study of this nature [28, 30]. Combined with the study aim, sample specificity, quality of dialogue in the interviews, and analysis strategy, this study’s sample size was considered to hold sufficient information power [40]. Data were coded by the first author (CE) and organized in Microsoft Excel. To overcome researcher biases and promote credibility, codes, themes and subthemes were determined through collaborative and reflexive processes between the three authors [41]. To support dependability and trustworthiness [42], four re-storied narratives with critical incidents and headlines were member-checked and subsequently discussed with the respective participants.

**Fig. 1:**
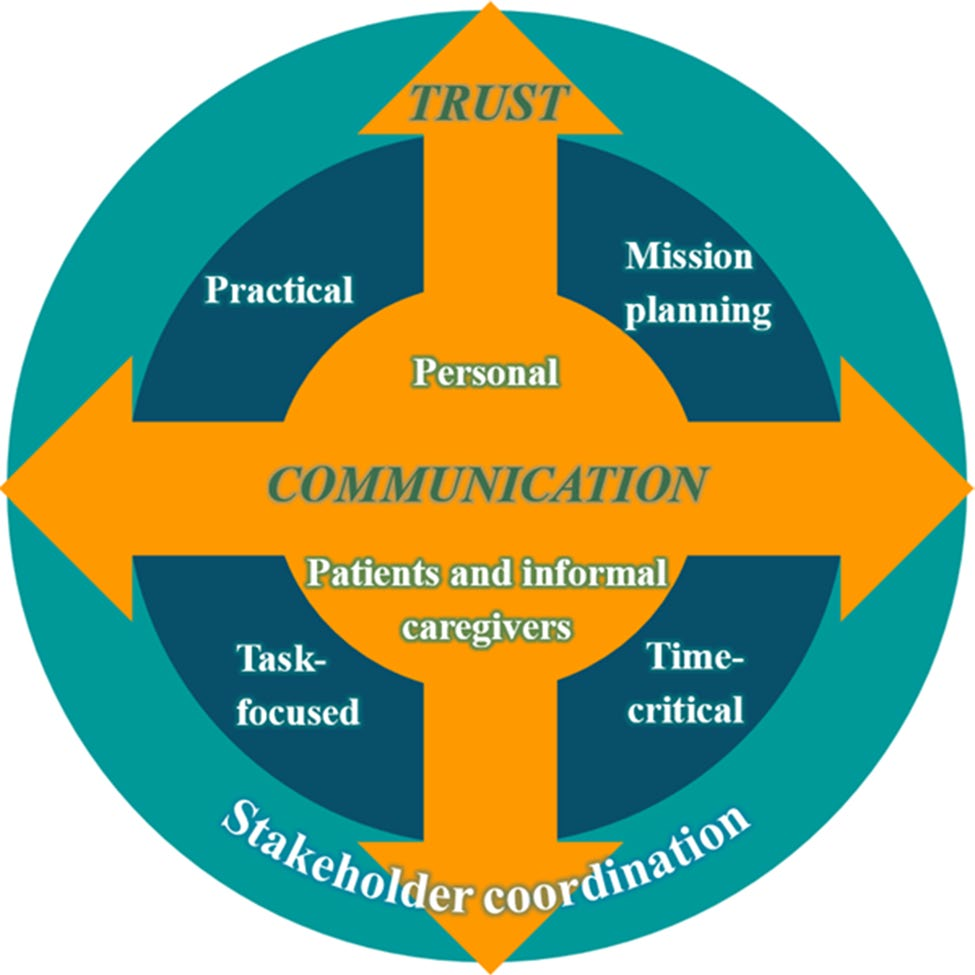
A model for successful adaptations during ambulance missions

In the cross-incident analysis, the meaning from across the twenty re-storied narratives was drawn out to identify core themes that represented the most important elements of successful adaptations. In this part of the analysis, the critical incidents from the re-storied narratives were congregated and diverged into data-driven themes [43], after which core themes were identified [29]. Initially, thirty-seven themes emerged, which were merged, split and relabeled ultimately forming seven core themes with sixteen adherent subthemes (see Table 1). The results will be presented as text descriptions with belonging quotes, and the main findings will be synthesized in a model (Fig. 1). The model was developed by arranging the core themes in relation to each other together with underlying features crossing all themes.

**Table 1:** Themes, subthemes and descriptions

Themes	Description	Subthemes
Adaptation in mission planning (CIs: 54)	Adaptations in mission planning beforehand of reaching the patient site and during mission revisions.	*Visualization - adapting focus and mental state* *Adapting preparatory initiatives*
Practical adaptations (CIs: 52)	Ad hoc adaptations contributing to problem solving, logistics, task performance and mission efficiency	*Transport - making the travel route as smooth as possible* *Creating adequate workspace* *Adapting the use of equipment*
Time-critical adaptations (CIs: 34)	Adaptations that saved time in patient transport, treatment, or both.	*Adaptations to reduce the time-to-treat* *Prioritizing measures based on criticality* *Adapting by deliberately deviating from protocol*
Personal adaptations (CIs: 98)	Individual adaptations based on the APs personal characteristics and traits, premises, and attributes.	*Adapting by exploiting personal abilities and skillsets* *Stress management*
Task-focused adaptations (CIs: 43)	Adaptations in collaborative decision-making processes between APs and their working partner.	*Adapting the workload between working partners* *Adapting measures based on clinical evaluations*
Adaptations in stakeholder coordination (CIs: 113)	Adaptations in collaborative processes with other stakeholders.	*Adapting task distribution in stakeholder collaborations* *Purposefully adapting communication for shared situational awareness*
Adapting to patients and informal caregivers (CIs: 58)	Adaptations that considered and involved patients and informal caregivers	*Adjusting levels of interaction with patients* *Adapting levels of informal caregiver involvement*

APs = Ambulance professionals, CIs = Critical incidents

### Reflexivity

Reflexivity was practiced with continuous, collaborative, and multifaceted research team reflections to evaluate how issues of subjectivity and context influenced the research process [42, 44]. The authors comprise a physical therapist (CE) with no previous knowledge or experience in prehospital services, although with substantial experience in conducting interviews in clinical practice. Reflexive notes were made after each individual interview with the participants. The two co-authors have extensive experience in health services research and safety sciences (KA) and prehospital critical care (SJMS). Having varied experiences and roles provided different perspectives. and thereby preconceptions, which were reflected upon and discussed throughout the research process. The analysis process was logged in reflection notes by the first author (CE) throughout the research process. The log contained information, reflections, questions for discussion, and the reasons for reaching consensus or making changes.

## Results

The analysis provided rich descriptions of APs’ successful adaptations across a broad variety of prehospital contexts, such as traffic accidents, cardiac arrests, or childbirth. Although participants were not explicitly asked to describe critical missions, most of them did. The adaptations are driven by various reasons, intentions, and goals, and differ in their levels of impact. The themes exhibit overlapping properties, reflecting the dynamic nature of prehospital services and the contemporaneous inward- and outward-directed reasoning and intentions that underpinned the adaptations. Themes, subthemes and descriptions are presented in Table 1.

### Adaptations in mission planning

Adaptation in mission planning involves adaptations that contribute to the APs preparedness before reaching the patient site or during mission revision, where further planning of mission execution is required. Ambulance cars are typically staffed by two APs, who formulate an initial plan for the missions while en route to the patient site. They relay information from the Emergency Medical Communication Central about the incident and initial emergency call but have limited access to the patient’s previous medical history. As a result, they rarely possess a complete picture of the patient’s condition, nor the situation they are entering. Consequentially, APs must adapt their planning to prepare clinical assessments, patient treatment, and their own actions individually, in collaboration with their working partner, or by utilizing other resources.

These adaptations were based on both information and experience, and were described as internal or individual adaptations, such as regulating one’s own mental and physical state, or adapting in collaboration with their working partner in practical preparation, problem framing, risk assessment, and resource coordination.

#### Visualization - adapting focus and mental state

The APs described visualization as mental processes of adaptation on their way to the patient site. These visualizations helped set expectations for the mission and guided their mindsets and strategic approach. They could include specific focuses, such as going through checklists and procedures, or considering plausible scenarios and solutions to potential challenges at the patient site by drawing on prior experiences. Visualizations were shared between working partners, helping them prepare for the unknown, both worst-case scenarios and the possibility that the situations might not be critical at all. The following is an example of individual visualization:

*The measures were to control my pulse and breath and think through the possibilities the situation might present when we arrived*,* such as if there were multiple patients*,* how I should triage*,* that I needed to create space to work*,* and so on.* (Nightclub shooting)

In this example, the AP adapted their mental state by internally creating a form of structure for the upcoming situation and context, which provided them to better be able to plan the mission. This subtheme overlaps with the theme ‘Personal adaptations’ but differs due to circumstantial factors and timeframes for visualizations to take place before- versus during patient treatment.

#### Adapting preparatory initiatives

Adaptations in mission preparation involved defining tasks and the primary goals of the missions, where plans were verbally articulated and coordinated en route to the patient site. This contributed to defining work frames and fostered a shared situational awareness, revealing the APs’ expectations, presumptions, and attitudes, thereby providing opportunities to adapt their mindsets before reaching the patient site (i.e., visualization). Risk assessments were conducted throughout the mission planning processes and were communicated out loud, which also involved self-assessment of their own skills and competencies. This open dialogue prepared them for the necessary adaptations at the patient site and supported practical application of skills in task distribution and team coordination. In the following example, the APs strategized a plan by initiating contact with appropriate resources for efficient patient evacuation, while simultaneously adapting to the public space, and maintaining the patient’s privacy:

*We planned to meet at a site that everybody knew where was*,* and where it was easy to get the patient ashore and into the ambulance*,* because there aren’t so many people there.* (Suicide attempt from bridge)

Preparations also included more proactive efforts to obtain patient information. This involved initiatives to gain access to the patient’s journals via other healthcare providers’ journal systems, requesting additional information about the patient from the Medical Emergency Communication Central, or involving informal caregivers or the patient’s regular healthcare providers in patient assessment.

### Practical adaptations

Practical adaptations were described as ad hoc measures, sometimes of a creative nature, that contributed to problem-solving, logistics, task performance, and efficiency. Practical adaptations demonstrate the APs’ ability to make use of “what’s at hand”, making small tweaks and adjustments of relevance to mission execution and quality. This theme overlaps with the theme ‘Adaptations in mission planning’ and reflects the participants’ field experience, providing opportunities for “thinking ahead”, and taking cautions preparing for the unexpected.

#### Transport - making the travel route as smooth as possible

Transport adaptations were made with considerations to safety, patient comfort, and efficiency in patient evacuation, transportation, and handover. Adaptations involved temporary, purposeful solutions such as strategic placement of the ambulance cars, clearing transportation routes of obstacles, using accessory equipment as supplementary safety measures, or adjusting driving patterns. In the following example, the APs recognized that evacuating the patient could be challenging. Therefore, they adapted by positioning the ambulance car to allow for easy access for the next ambulance and to minimize the distance the patient needed to be carried during evacuation:

*We parked right outside*,* with enough space around the car for the next ambulance that was to arrive*,* and with patient evacuation in mind.* (Cardiac arrest in obese patient)

In another mission, the APs contacted the receiving hospital ward to have them remove all obstacles, ensuring a clear pathway upon arrival:

*We asked the department at the hospital to clear the hallway for patients and equipment that might be in the way*,* so that we could go straight up*,* without exposing the dead patient to other patients.* (Cardiac arrest in child)

#### Creating adequate workspace

APs described how they created adequate workspace to be able to perform their tasks efficiently. They adapted to their surroundings or modified the surrounding structures to meet their operational needs. For example, they strategically positioned themselves or equipment, or physically moved patients or furniture to create more room:

*We took a few extra seconds to move things around*,* so that we better could work on the patient*,* and placed the equipment where everyone could see the screens and have full access to the patient.* (Cardiac arrest in apartment)

Another example of adapting their workspace involved moving the patients to the ambulance car, where equipment was readily available in case the patient’s condition deteriorated, such as in high-impact accidents (e.g. a car crashes) with potential for undetected internal bleeding.

#### Adapting the use of equipment

Equipment adaptations provided workload relief, increased the APs capacity to perform necessary tasks, and contributed to consistency and safety in mission execution. Adaptations included using assistive technological equipment for assessments, treatment and information transfer, or using non-technical equipment as preventive measures, for example, using tape to ensure that needles, wires or tubes stayed in place. In the following example, the APs adapted their workload by using a mechanical heart compression device (LUCAS), which freed them to initiate and perform other necessary tasks for effective mission progression:

*When we got the LUCAS on*,* we were free to get off the cold ground and rather zoom out and get a grasp of the bigger picture and focus on getting the patient out.* (Diving accident)

Based on their plans, APs made sure all necessary equipment, such as gloves, headlights, and equipment intended for patient assessments and treatment, was ready and easily accessible. They often included more equipment than strictly necessary “just in case,” which increased their opportunities to adapt to various scenarios:

*We knew the delivery of the baby could be critical*,* but nothing indicated that it should be so… Luckily*,* we had gotten the suction and bag with the mask ready. *(Childbirth in ambulance)

### Time-critical adaptations

The participants described time-critical adaptations that contributed to time efficiency relevant to mission progression and were crucial for patient outcome. As opposed to the theme ‘Practical adaptations’, time-critical adaptations did not include elements of creativity and small tweaks, but rather reflecting the mission’s level of urgency and the APs ability to make good decisions under pressure. Adaptations involved driving route planning, workarounds, making quick decisions, and prioritizations, and logistical time-saving measures based on clinical criticality or having the upcoming part of the mission in mind.

#### Adaptations to reduce the time-to-treat

In cases of long travel distances or delays before other health care providers arrived, timely measures were necessary to reduce time-to-treat. Adaptations included reducing the distance to the next resource by transporting the patient towards the approaching unit or optimizing logistics, making the mission execution more practical by sharing or handing over responsibilities and tasks. For example:

*There was no time to waste. While we waited for the doctor to arrive*,* we got the patient on a chair and carried her down the stairs immediately.* (Multimorbid patient with chest pain)

In this mission, the patient went into cardiac arrest before the physician arrived. The APs adapted by evacuating the patient to meet the physician in the ambulance car, allowing advanced treatment to begin during patient transport.

#### Prioritizing measures based on criticality

Adaptations were typically made in high-urgency situations and reflected the APs’ ability to recognize, analyze, make quick decisions and act accordingly. Adaptations involved conscious trade-offs, where the foremost clinical or time-critical measures were prioritized, for example to not prioritize pain management in life-threatening situations because there was no time to wait for its effect:

*There was no time for pain medication*,* I just had to start filling the gunshot wound with gauze or the patient would bleed out.* (Shooting in nightclub)

Another example demonstrates the APs’ ability to seize opportunities when they occur and adapt prioritizations accordingly:

*The patient was close enough to the riverside for the rope to reach. We had to act immediately. We knew the other ambulance was on the way*,* so my partner broke communication with the Emergency Medical Communication Central and threw the radio on the grass*,* so that she could assist me with both hands.* (Suicide attempt from bridge)

This example illuminates the innate trust in between working partners, and other APs, which served as an element of security in the adaptation of breaking communication with the emergency medical communication central.

#### Adapting by deliberately deviating from protocol

Deliberately violating protocols were rare but an important part of time-critical adaptations. Such adaptations were well-intentioned and crucial for patient outcome, and grounded in the APs’ professional ethos, in combination with extensive occupational experience and clinical knowledge. For example:

*It was impossible to ventilate using the mask*,* so I kept it simple and gave mouth-to-mouth to get air in the lungs as an emergency solution. That worked.* (Childbirth in ambulance)

### Personal adaptations

The participants described adaptations that were founded in their personal traits, premises, and abilities. The adaptations involved personal characteristics, such as confidence, adaptability and mental strength, and the ability to utilize their skillsets and life and work experience in advantageous ways.

#### Adapting by exploiting personal abilities and skillsets

The APs described adaptations that involved utilizing their personal skills, experiences and self-assessment abilities to handle mission challenges, such as taking advantage of their climbing or swimming skills. They drew on experience and knowledge from their personal life and applied these in beneficial ways, for example, in situations involving care for the patient’s family or using knowledge about local geography and stakeholders to self-organize missions, thereby creating opportunities for creative ad hoc solutions. This subtheme had the intrinsic feature of adaptability, where the APs demonstrated the ability to quickly adapt, reset or change their plans or mindsets in response to unexpected situations when they were caught off guard. Personal abilities became more refined with work experience and increased concurrently with the APs’ levels of confidence in their own skills and competence. An example of personal adaptation is as follows:

*I have two daughters myself and talked to the patient’s daughter as I would talk to them. No shouting or yelling*,* but with a kind and calm tone of voice.* (Cardiac arrest with child present)

Another example of personal adaptations demonstrates the APs’ ability to easily shift their mindset when the initial plan required reframing:

*When we arrived*,* the patient was much sicker than we had anticipated based on the message from the Emergency Medical Communication Central. If he hadn’t spoken or moved a bit*,* I would have thought he was dead. The mission callout was initially to transport him to the hospital*,* but we had to start treatment right then and there.* (Cardiac arrest in doctor’s office)

In this example, there was no doubt in the APs’ minds as to whether patient treatment could wait. Their confidence and swift shift in focus also influenced the other healthcare professionals and APs on-site, helping them coherently adapt to the new plan.

#### Stress management

Stress management adaptations were described as strategic adjustments and measures directed both inward and outward, with the purpose of facilitating inner calmness for themselves or calming the situation at hand. Inward stress management adaptations were made through internal self-assessments and involved taking a few seconds to mentally reflect on their present emotional state and adapt to a more advantageous mindset to increase their capability to handle the situation. This included changing or blocking their negative thought patterns, setting emotions aside to be dealt with later, or choosing to ignore disturbing factors from the surroundings. An example of such stress management is:

*I set my ego aside*,* and didn’t exclude any parts of the clinical examinations*,* even though I thought the informal caregiver was a bit pushy and the patient’s symptoms were vague. The ECG showed that the patient had a heart attack.* (Multimorbid patient with heart attack)

Stress relief techniques, such as adjusting their breathing, pulse, and muscular tension were described as measures that contributed to reduce their body’s physical state of stress, making it easier to remain patient- and task-focused and better handle external stressors. “Falling back” on protocols, training and foundational skills was also an adaptation that contributed to stress management, particularly in chaotic or highly critical situations.

Outward stress management adaptations were purposefully directed towards patients, informal caregivers, working partners, or other stakeholders to de-escalate conflict, agitation, or distress. These adaptations were described as changing their tone of voice and wording or using other communication strategies such as body language or adapting their presence and availability without communicating it out loud.

### Task-focused adaptations

Task-focused adaptations involved adaptations made in collaborative decision-making processes between APs and their working partner. They were described as measures based on evaluations and prioritizations, aimed at maintaining focus and follow through with the most important tasks of the mission with high quality. This theme overlaps with the theme ‘Personal adaptations’ as they both comprise the APs’ skillsets and stress management but differs as task-focused adaptations also involve their working partner.

#### Adapting the workload between working partners

Participants described workload adaptations as sharing or distributing tasks between APs and allocating or rotating ambulance personnel to maintain high-quality task performance. An example is:

*When he had performed heart compressions for a while*,* I saw that he was getting tired*,* and told them to swap*,* so that the compressions continued to be good enough.* (Trauma accident)

Adaptations were based on logistics or the APs’ capacity or skillsets, where the total workload was fractionated, preventing APs’ from having to manage multiple tasks simultaneously:

*We chose to only bring the rope with us down to the water*,* because we knew others soon came to assist us.* (Suicide attempt from bridge)

Workload adaptations also involved awaiting task initiation or handing over responsibilities to APs with the appropriate capacity or competence, in order to avoid unnecessary risks that could compromise patient safety. This subtheme also reflects the trust APs have in each other’s team efforts and competences.

#### Adapting measures based on clinical evaluations

Adaptation in clinical evaluations involved performing tasks in alternative ways, repeating clinical assessments to verify findings, or awaiting initiative, such as exercising caution administrating medication:

*We could have administrated more muscle relaxers*,* but when the patient was hypothermic*,* we had to take caution regarding the dose.* (Ancle fracture-patient found outside)

These adaptations reflected the APs’ practical knowledge, experience, and their ability to “pause” the moment or take a step back to conduct a more comprehensive evaluation:

*I awaited the nitro*,* because I was not able to measure the patient’s blood pressure properly.* (Cardiac arrest)

### Adaptations in stakeholder coordination

Adaptations in stakeholder coordination involved initiating outward coordination with actors within the emergency services, such as police and fire departments, as well as other health resources like midwives, hospital departments, and nurses. These adaptations contributed to shared situational awareness, and to logistical and practical solutions that made mission planning and execution run more smoothly and effectively. Adaptations facilitated stakeholder involvement and team efforts in decision-making processes, much like in the theme ‘Task-focused adaptations’. However, a substantial difference is that this theme involves adaptations to stakeholders with other premises and/or motives than the APs.

#### Adapting task distribution in stakeholder collaborations

Adaptations were described as initiatives made by the APs during mission coordination and execution, which contributed to effective and positive collaboration between stakeholders. The APs made adaptations in synergy with other stakeholders’ actions, taking initiatives for tasks and assignments to be shared across the different stakeholders’ occupational frames of reference and “working spheres”. Adaptations could include asking firefighters or police officers to do certain tasks for them, as demonstrated in the following examples:

*We asked one of the police officers to drive the ambulance*,* so that the both of us could work on the patient. The police officer immediately obliged and asked: ‘where to?’.* (Suicide attempt from bridge)

*When the fire fighters came*,* we asked them to prepare the gurney*,* and told them where to place it and lay a blanket and carrier on it*,* so that it was all set for evacuating the patient.* (Trauma accident)

APs also adapted by adequately adjusting their own roles to fit the needs of the situation, such as functioning as assistants to other occupational groups, typically in handover situations where specialized resources had primary responsibility for the patient:

*As soon as we arrived at the hospital ward*,* the ER nurses in the trauma team re-intubated the patient right there in the hallway. My colleague paused and started the lung compression machine each time they told us to. This made the handover more effective.* (Suicide attempt from bridge)

#### Purposefully adapting communication for shared situational awareness

Adaptations in communication were substantial in stakeholder coordination and were described as essential for effective and safe mission execution and progression, both with the Medical Emergency Communication Central and with stakeholders on-site. Adaptations were based on occupational experience, familiarity or unfamiliarity with working partners and stakeholders, and knowledge about “the system”. The descriptions of adaptations portrayed the APs’ communicative adaptability, where what, how, when, why, to whom, and to what extent things were communicated was purposefully adapted to create shared situational awareness and facilitate initiatives from-, and collaboration with others. Concrete examples included thinking out loud, speaking with authority, directly addressing someone, or double-checking whether things were heard and properly understood. More subtle adaptations involved non-verbal communication, such as looks, gestures and body language. These adaptations were especially prominent in situations that called for high focus, or when discretion was needed, for example, when family members were present:

*Mostly*,* we “spoke” non-verbally*,* and the tasks were distributed by use of glances and nods.* (Shotgun suicide)

Persistent communication of a persuasive nature aimed to facilitate understanding, and was based on the APs’ confidence, knowledge and experience. Adaptations included explaining or arguing their case, asking questions to support reflection, or seeking others’ opinions to build a strong case. These adaptations were typically necessary in situations where there was a misalignment in situational awareness and understanding between APs and off-site stakeholders, such as the Medical Emergency Communication Central, which consequentially could lead the APs to coordinate the mission independently in collaboration with other APs, health care providers and stakeholders on-site:

*Based on their guidelines*,* the Emergency Medical Communication Central told us to take the patient to the hospital*,* but the patient would be better off being treated with antibiotics at the care home. Since they wouldn’t listen*,* I said*,* ‘you know what*,* I will call someone else*,* because this isn’t working’*,* and we called the patient’s medical doctor directly and explained the situation*,* who then arranged for the antibiotics to be sent in a taxi to the care home.* (Lung infection in patient with dementia)

The same occurred in situations where the APs *knew* that their needs would not be accommodated by the Medical Emergency Communication Central, so they deliberately chose not to initiate contact in order to save time. These adaptations were carefully considered based on clinical assessments, and when time was too critical to spend on negotiation:

*We chose not to call the hospital*,* because they usually say no when it comes to patients like that. They would have told us to ask the emergency doctor to come to us instead*,* but that would have taken hours*,* and the patient would most likely have had a cardiac arrest by then.* (Multimorbid patient with chest pain)

Adaptations in stakeholder coordination illuminated the relational aspects of multi-stakeholder collaborative processes, and how stakeholders adjust to one another.

### Adapting to patients and informal caregivers

This theme overlaps with the theme ‘Adaptations in stakeholder coordination’ but differs as patients and informal caregivers are not a professional contributing resource. Adapting to patients and informal caregivers involved APs adapting their approach and way of communicating to obtain information and achieve collaboration. Adapting to informal caregivers also involved efforts to include them as a resource for practical tasks. These adaptations demonstrated the fine line between balancing clinical demands and respecting the patient’s and informal caregivers’ autonomy.

#### Adjusting levels of interaction with patients

Adjusting patient interaction was described as taking considerate measures in communication as well as efforts to create understanding, collaboration and mission progression based on the situations at hand and the patient’s autonomy.

The descriptions demonstrated the APs abilities to comprehend and relate to the patient’s situation and act according to their moral and ethical responsibilities when handling people in vulnerable situations, while simultaneously tending to clinical demands, as demonstrated in the following example:

*We talked to the patient about what was going on. Even though he didn’t respond we should not treat him as if he was deaf.* (Transporting terminally ill patient)

Some situations called for limited patient involvement, such as when the patient had withdrawn from the situation because they did not have the mental or physical capacity to engage:

*When we offered to help*,* she clearly wanted to be left alone and was not interested in us helping her more than utterly necessary*,* which I understood*,* so we let them be.* (Premature baby born in the ambulance)

Sometimes there was no time to include the patient due to the situation’s criticality, while in other cases, a high level of patient involvement was necessary, typically in situations where patients were resistant or in distress.

#### Adapting levels of informal caregiver involvement

Adapting by involving informal caregivers in clinical assessment contributed to provide information about the patient’s baseline condition and could reveal relevant changes or deviations. Adapting by including informal caregivers as a resource during the mission, such as assigning them tasks or giving instructions, provided them with a sense of purpose in contributing. At the same time, it gave the APs “more hands on the job” and reduced disturbances or interruptions from the informal caregivers, as they were occupied:

*I gave the bag of fluid to the patients’ boyfriend so that he had a specific task he could do to help. He then stopped pacing around and interrupting us.* (Patient jumped from building)

APs also described adaptations regarding how they handled informal caregivers by creating a safe space and setting expectations they could adhere to. This established structure and helped keep the situation composed. Adaptations involved adjusting the level of availability and support or alternatively involving other stakeholders for support. Keeping informal caregivers “adequately informed” at an appropriate time involved adapting information flow and timing when information was provided. This sometimes meant preventing or delaying information from being communicated until the informal caregivers were in an environment with the necessary support available, typically in highly sensitive situations such as suicide or child death:

*I closed the ambulance door every time I entered or exited*,* and took the mother aside a bit so that she could not see into the ambulance. It’s not something a mother should see… We drove to the hospital before informing the parents that their child did not make it.* (Child death)

In summary, the results demonstrate that several themes and subthemes had overlapping properties, reflecting the complexity of prehospital services, and the breadth of contextual and collaborative influences on the adaptations made by APs. The most prominent overlapping subtheme was ‘Purposefully adapting communication for shared situational awareness’ which overlapped across all types of adaptations within the realm of collaborative processes during missions. Communication is an intrinsic feature of ambulance missions as stakeholder coordination is a major prerequisite for their success. Trust, in the form of reliance in partners, confidence in task distribution and responsibilities, and interdependencies among stakeholders, is an underlying feature for successful adaptations in ambulance missions. This is illustrated in Fig. 1, displaying the different types of adaptations in relation to each other, along with the two foundational features of communication and trust, highlighting the inherent relational nature of ambulance missions to which APs must adapt.

The outer circle represents ‘Stakeholder coordination’, which in various ways, encompasses all adaptations of multi-stakeholder collaborative processes. The middle circle represents adaptations among stakeholders at a functional level of mission performance, and the themes in the center, reflects individual and non-professional relational adaptations. The arrows represent the intrinsic elements of trust and communication vital for all adaptations across the collaborative relationships of ambulance missions.

## Discussion

The results describe a wide range of successful adaptations made by APs during ambulance missions, from the most mundane to highly creative ones. A deeper insight into adaptations can contribute to a better understanding of what makes things work, which consequently can reveal factors that impact decision-making processes and other prerequisites for successful ambulance missions.

Other studies on adaptations in hospital contexts such as intensive care units, have identified adaptive strategies for responding to everyday pressures, such as increasing the competency level of incoming staff, canceling surgeries, or adjusting the schedule for surgery theatres [20, 22]. These strategies are organizational, and not directly applicable to the prehospital context as, APs do not have the same possibilities to reorganize during mission planning or patient treatment. This study contributes with understanding of contextual factors in prehospital services, such as structural and social working environment and the levels of variability that APs must adapt to, thereby providing valuable insight for organizational development.

In a study on organizational factors influencing clinical reasoning for APs, Andersson et al. found that several factors negatively impacted APs’ work environment and mission planning and performance, such as a broad range of communication challenges, insufficient equipment and guidelines, and vague structures and formalities regarding responsibilities. APs were for example, expected to conduct proper assessment but would still be overruled by other actors with different situational views and motives [45]. To overcome these barriers, our study documented a set of adaptive abilities in the everyday clinical practice of APs, ranging from personal (e.g., exploitation of skillset) to team (e.g. workload adaptation), to organizational factors (e.g., stakeholder coordination).

The adaptations portrayed the APs ability to make considerations that exceeded beyond their own actions in response to a particular challenge or change in collaborative processes, by also considering the needs of other stakeholders, patients and informal caregivers, and suitably incorporating or eschewing them during missions. This highlights the APs’ ability to balance interpersonal encounters with the demands of medicine and care, reflecting competencies that may impact patient safety [14, 46]. Since the study’s aim was to describe adaptations considered successful from the APs’ viewpoint, caution is warranted, and more research is needed to address whether these adaptations are also considered successful by other stakeholders.

Adaptations, trade-offs and workarounds are essential in ambulance missions, but they can also mask underlying system challenges or dysfunctions, which can leave the system and its managers with false impressions of what works or not [11, 47]. Particularly two examples from this study illustrates such challenges, where conflicting understandings, needs and motivation between stakeholders were misaligned. These examples underscore issues related to clinical decision-making, risk perception, and non-conveyance [3, 48, 49]. In one of the examples, the AP’s needs were neither understood nor met, leading the AP to adapt by ignoring the Medical Emergency Communication Central’s instructions due to excessive resistance from the operator because of their adherence to protocol. In the second example, the AP, having experienced repeated rejection from the hospital, adapted by deliberately choosing not to initiate any communication with the hospital in the decision-making process. Although these adaptations were successful, system dysfunctions remained masked, missing opportunities for learning and system improvements. The Medical Emergency Communication Central serves as an invaluable resource in prehospital services, where mission coordination and communication between stakeholders are generally highly functional. However, the need for adaptations may still arise because of constantly changing team compositions, interprofessional differences in expectations, culture and education, perceived and actual hierarchies and power differences, and the episodic nature of teamwork [50].

There is a distinct difference between adaptations that occur in communication with stakeholders on-site compared to off-site. On-site adaptations emphasize the importance of relationships and shared situational awareness in collaborative decision-making processes. The extensiveness of communicative adaptations described in this study underscores that stakeholder coordination and collaboration in prehospital services deserves more focus in research [51], particularly regarding organizational cultures, hierarchical attitudes and possible friction [52].

This study demonstrates that trust is an underlying feature for successful adaptations. Ambulance missions comprise social relationships between stakeholders that rely on each other for effective and safe mission performance. Decision-making processes are, to varying degrees, always a team effort. Sharing experiences of uncertainty with others can improve decision-making processes and the ability to cope with difficult and non-routine situations [16]. Creating safe spaces for stakeholders to reflect collectively on their performance can foster a shared understanding of the local context, which, in turn, helps build collective trust [53, 54]. In cases where shared understanding of the local context between stakeholders were not reached during the missions, APs described taking initiative to debrief immediately afterward to discuss misalignments of understanding, reasoning and actions. Participation in such critical reflection contributed to build stronger relational bonds and facilitated learning through practice [55, 56].

This study adds value to the prehospital research field by providing insight into APs’ decision-making processes and the reasoning behind adaptations. These insights can be utilized by APs, management, and other stakeholders to support and improve ambulance missions and enhance collaborative processes in prehospital services.

## Limitations

This study uses CIT methodology to collect and analyze data, solely exploring successful adaptations. Some CIT methodology literature includes “wish list” items [32], where participants are asked to elaborate on potential items or issues that could have been helpful in the activity, but were not present. We deliberately chose not to include questions that sought to reveal such items as we wanted to explore the APs lived experiences. CIT is a retrospective methodology, where the aspect of credibility can be questioned due to the risk of poor memory recall and memory reconstructions. This risk was minimized as the participants were asked to share a self-selected mission that had made particular impact on them [28, 30]. To reinforce trustworthiness, participants were also involved throughout the data collection and analysis processes, making sure that the data was properly understood and presented.

This study was conducted in two health trusts in Norway, and the results transferability to other national regions or countries with different prehospital services and systems can be considered a limitation. No study can provide findings that are universally transferable [42], however, the findings can be presumed applicable to other prehospital service contexts, as the APs descriptions of adaptations might share similarities with parts of the world with different, but equally challenging conditions.

## Conclusions

The APs rich descriptions of ambulance missions have provided insight and new knowledge about successful adaptations in the prehospital service. The results demonstrate the APs ability to beneficially utilize their experience, knowledge, skillsets, creativity and personal attributes to make adaptations that contribute to safe and effective mission performance. Communication was the overall predominant feature of successful adaptations, which concurrently influenced stakeholder coordination and decision-making processes impacting team efforts and mission success. Trust was the dominant underlying feature of APs adaptations. Researching the role of contextual factors in adaptations such as differences in stakeholder perspectives, institutional boundaries, available resources, and surroundings provides opportunities for learning and system improvements. Further exploration of stakeholder coordination could provide insight into how cross-occupational and cross-stakeholder collaborative processes contribute to successful ambulance missions.

## Supplementary Information

Below is the link to the electronic supplementary material.

Supplementary Material 1

## Data Availability

The datasets analyzed during the current study are not publicly available as they contain information that could compromise the privacy of research participants but are available from the corresponding author on reasonable request.

## Abbreviations

APs: Ambulance professionals
AST: Ambulance service technician
CIT: Critical Incident Technique
