# Supplementary material for: Ambulance professionals’ adaptations in prehospital services: a critical incident study
Source: BMC Emerg Med. 2025 Aug 15;25:154. doi: 10.1186/s12873-025-01309-6 (PMC12357368; doi:10.1186/s12873-025-01309-6)
Supplement: Supplementary file 1 — Supplementary Material 1 [file 12873_2025_1309_MOESM1_ESM.pdf]

Study: Ambulance Professionals Adaptations in Prehospital: Care A Critical Incident Study

Interview Guide:
Individual Interviews with Ambulance Professionals in Prehospital Care

The purpose of the individual interviews is to gain insight into each participant's experiences related to adaptations in daily practice that contribute to achieving good results. How they learn and use experience/knowledge as part of the adaptations will also be included. The information letter is reviewed with the participants and the consent form is signed before the interview.

Opening Questions

- Can you tell me about yourself and your daily tasks?
- Can you tell me about the station/ambulance service you work at/in?
- Who do you work with and how long have you worked together?
- What are your daily routines?

Theme: Adaptations in Practice

Main question: Can you talk about a mission that you found successful? Describe the event from start to finish and try to include as many details as possible about what happened. Remember that it should be an event you think was carried out well based on what the situation required.

Potential follow up questions:

- When and where did it happen and what were the surroundings (weather, road conditions, time of day, etc.)? What significance did the time and place of the event have for the mission?
- What equipment was used and why exactly this?
- Was there something that happened with the patient that you had not experienced before?
- How did you experience the cooperation between you and your working partner? Was it a new partner or did you know each other well beforehand?
- Who else was present and did you know them beforehand? What were their tasks?

- How did you experience the communication between you, your partner, and the others?
- What significance did trust have for the way you worked and the choices you made (internally in the ambulance team and externally with possibly others involved)?
- What adaptations did you make during the event (e.g., tasks, consideration for the patient, communication, procedures, etc.)? What adaptations did others make? Were these adaptations you or others were used to making or did you/they improvise? What solutions did you come up with?
- What factors do you think were the most important for the event to be successful and what could have been done differently for further improvements?
